# Complementary and Alternative Medicine Use and Latina Breast Cancer Survivors’ Symptoms and Functioning

**DOI:** 10.3390/healthcare4040080

**Published:** 2016-10-31

**Authors:** Christina L. Rush, Tania Lobo, Adriana Serrano, Maxie Blasini, Claudia Campos, Kristi D. Graves

**Affiliations:** 1 Lombardi Comprehensive Cancer Center, Georgetown University, Washington, DC 20007, USA; christina.rush@ucdenver.edu (C.L.R.); Tania.Lobo@georgetown.edu (T.L.); Adriana.Serrano@georgetown.edu (A.S.); mmb300@georgetown.edu (M.B.); 2 Nueva Vida, Inc., Alexandria, VA, 20036, USA; ccampos@nueva-vida.org

**Keywords:** complementary medicine, alternative medicine, breast cancer, quality of life, Latina, religious beliefs, spirituality, prayer

## Abstract

Complementary and alternative medicine (CAM) is used widely in cancer populations, particularly among women, and has shown promise for addressing symptom and functioning outcomes. Few studies to date have evaluated CAM use and associations over time with symptoms and function among Latina breast cancer survivors. We administered a baseline (*N* = 136) and follow-up (*n* = 58) telephone survey in Spanish or English assessing Latina breast cancer survivor demographics, physical function, anxiety, depression, fatigue, satisfaction with social roles, and both CAM activities and devotional and spiritual practices. About one-third of our sample (35% baseline; 36% follow-up) reported using CAM (yoga, meditation, massage, or herbal/dietary supplements). We assessed devotional and spiritual practices separately from CAM (church attendance, prayer, religious groups, and reading devotional and religious texts); the majority of Latina survivors reported devotional and spiritual practices (80% baseline; 81% follow-up). At baseline, CAM demonstrated a positive association with better physical functioning and lower depression. In contrast, CAM use at the time of follow-up appeared to be related to lower levels of satisfaction with social roles and physical function. In longitudinal analyses, devotional and spiritual practices at baseline significantly predicted lower anxiety, depression, and fatigue at follow-up. Findings suggest CAM plays a complex and not always linear role in symptoms and function outcomes for Latina breast cancer survivors. These findings contribute to the literature on longitudinal CAM use and associations with symptom and functioning outcomes among Latina breast cancer survivors.

## 1. Introduction

Complementary and alternative medicine (CAM) is defined in terms of its relationship to conventional medicine. If used alongside conventional medicine, a CAM practice is considered “complementary”; if used to replace conventional medicine, a CAM practice is considered “alternative” [1]. For the purposes of this study, CAM includes iterations of mind-body medicine (yoga, meditation), massage, and naturopathic medicine (herbal supplements and dietary regimens). Aspects of religious and spirituality practices (church attendance, prayer, religious groups, and devotional and religious texts) have been considered part of CAM in some prior studies [2,3]; other work calls for a distinction between CAM and spirituality and devotional practices [4]. CAM is used widely in breast cancer populations [5,6,7], and randomized controlled trials (RCTs) of specific CAM modalities such as yoga [8,9] and mindfulness-based approaches [10], among others, have shown initial promise to reduce symptoms and improve function related to quality of life (QOL) [11].

### 1.1. Current Literature

Research has begun to explore CAM use among minority breast cancer survivors, including Hispanic/Latino women, one of the fastest growing minority groups [12]. In the limited number of studies examining CAM use exclusively among Hispanic breast cancer survivors, rates of CAM use appear relatively high (e.g., ranging from 65% to 93% depending on CAM modality; [13,14,15]). Studies with larger but more heterogeneous samples of non-Hispanic white and racial and ethnic minority breast cancer survivors illustrate a similar enthusiasm for CAM [3,9]. For example, in a study with Latino, black, white, and Chinese breast cancer survivors, nearly all participants perceived CAM as helpful and would recommend it to their friends [3]. Evidence from these prior studies suggests that Latina survivors who used CAM are more likely to have higher education and income levels, are of younger age, have private insurance, exercise, or have attended cancer support groups [3,9,11,14,15]. Among CAM modalities, Latinas most often reported using dietary therapies and spiritual healing to help manage a breast cancer diagnosis [3]. Prayer and other spiritual practices appear salient for many cancer survivors [16,17,18,19], with one prior study reporting that 62% of cancer survivors used prayer [19]. Other research has documented religion and spirituality, including prayer, as important to Latina breast cancer survivors’ coping efforts and health-related QOL [20]. Some survivors, including Latinas, appear to view CAM modalities as potential “cures” for breast cancer, although these findings are mixed in the literature [11,21,22]. In the present study, we distinguish between CAM and spiritual and devotional practices because of the importance of spirituality and religion among many Latinas and to provide greater specificity to our understanding of how these factors relate to cancer survivors’ symptoms and functioning [4,16,20].

### 1.2. Gaps in the Literature and Study Purpose

We are aware of only two studies in the literature that examine CAM exclusively among Hispanic/Latina breast cancer survivors [14,15]. Moreover, few studies of CAM use in race/ethnic minority women with cancer have included a longitudinal research design to examine associations between CAM and symptoms and functioning outcomes over time. The purpose of this study was to examine the frequency of CAM use in Latina breast cancer survivors and identify associations of CAM use with patient-reported symptoms and functioning outcomes over time. For this study, we define symptoms and functioning as those identified in prior literature as important to breast cancer survivors: physical function, satisfaction with participation in social roles, anxiety, depression, and fatigue [23].

## 2. Materials and Methods

### 2.1. Participants

In partnership with four community-based organizations (CBOs), we recruited breast cancer survivors who self-identified as Hispanic or Latina from 2014 to 2016 (see Section 2.2.1). Participants were recruited as part of a larger parent study involving Latina breast cancer survivors and their caregivers. In the parent study, we conducted a randomized controlled trial to improve QOL for Latina breast cancer survivors and their caregivers. Survivor-caregiver pairs were assigned by chance to the intervention or usual care. In the present study, we focus on survivors who were assigned to the usual care condition. Participants in usual care could receive any of the typical support, navigation, or information services at the four collaborating community-based organizations (see below for additional information about usual care and Rush et al. (2015) for a more detailed description of the larger study and intervention) [24]. Potential participants were informed on-site about the study by recruitment staff. Participants provided verbal or written consent to the recruiter to share their contact information with the bilingual Spanish-English study interviewers at the Lombardi Comprehensive Cancer Center at Georgetown University. To be eligible, participants had to be English- or Spanish-speaking Latina breast cancer survivors, between 18 and 85 years of age and, for the parent study, able to identify a primary caregiver. Latinas diagnosed at any stage of disease or any amount of time since diagnosis were eligible. Participants received gift card compensation at the baseline ($10) and follow-up telephone assessments ($15).

### 2.2. Procedures

**Figure 1:**
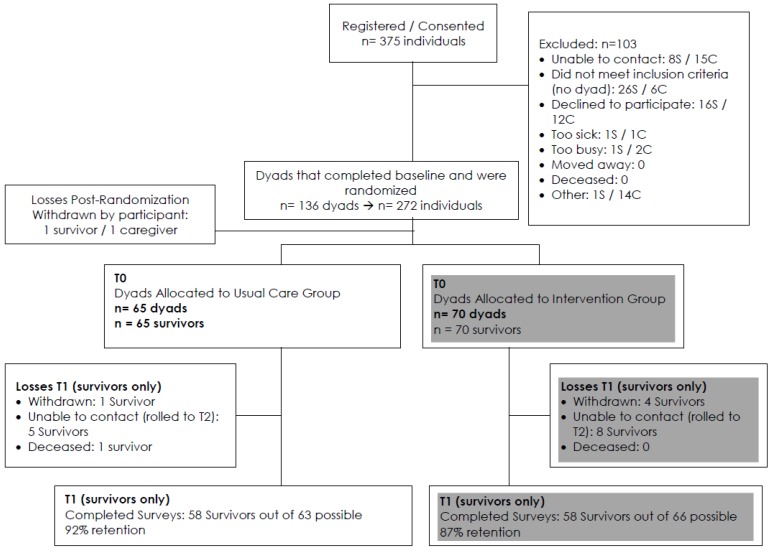
Nueva Vida Intervention. Notes: S = survivor; C = caregiver. Usual care (UC) follow-up response rate is 58/65, or 89%. However, obtainable response rate is 58/63, or 92% (one UC survivor died; another withdrew).

Participants completed assessments at baseline and an initial follow-up conducted approximately 4 months post-baseline; we include follow-up outcomes from breast cancer survivors only in the usual care group (*n* = 58). The sample of 58 at follow-up represents 89% (58 out of 65; see retention note below Figure 1) of survivors who were eligible for follow-up in the subsample we focused on for the present analyses. Specifically, we elected to focus on changes over time among Latina survivors in the “usual care” condition that was part of a larger intervention. Follow-up assessments for the present sample of participants coincided with the timing of the intervention group follow-up assessment. We elected to pursue follow-up analyses only with participants assigned to usual care to ensure that results were not influenced in any way by the parent study intervention. The CONSORT diagram “Nueva Vida Intervention” (Figure 1) identifies the numbers of eligible participants at each time point.

Usual care included a range of support and information services across the four CBOs such as: support groups, educational lectures, workshops, social events, screenings, navigation programs, and referrals to low-cost or free services.

We conducted surveys in Spanish or English according to the participant’s language preference, and collected survey responses using REDCap (Research Electronic Data Capture), which is a secure electronic data capture tool [25]. Study procedures were approved by the MedStar-Georgetown Oncology Institutional Review Board.

#### 2.2.1. Participating Organizations

Four CBOs partnered with Georgetown University in the conduct of this study: Gilda’s Club NYC (New York, NY, USA), Latinas Contra Cancer (San Jose, CA, USA), Nueva Vida, Inc. (Alexandria, VA, USA), and SHARE (New York, NY, USA). These CBOs serve Latina cancer survivors and their families from the Caribbean and North, Central, and South America, providing support and care in a linguistically and culturally sensitive way.

### 2.3. Measures

Measures in the current study included the following and are described in Section 2.3.1, Section 2.3.2 and Section 2.3.3:

#### 2.3.1. Demographic and Clinical Variables

Demographic and clinical variables included survey language, race, marital status, age, time since breast cancer diagnosis, stage at diagnosis, education, employment, income, insurance, marital status, years in the United States, and treatment type.

#### 2.3.2. Patient-Reported Symptoms and Function

We measured patient self-reported symptom and functional outcomes using five short-form measures from the Patient-Reported Outcomes Measurement Information System^®^ (PROMIS^®^; HealthMeasures, Northwestern University, Chicago, IL, USA) (see Table 1) [26,27,28]. We measured: physical function (PF 6b), satisfaction with social roles and activities (“Social Function”; SF 6a), depression (Depression 6a), anxiety (Anxiety 6a), and fatigue (Fatigue 4a). PROMIS measures were scored using the Assessment Center scoring service [29]. The scoring service generates a score (50 = U.S. general population [30]; 10 = one standard deviation) for each measure using a standard expected a posteriori (EAP) estimation procedure for individual response patterns [31]. PROMIS measures have been translated and validated for U.S. Spanish-speaking cancer patients [28,32]. PROMIS measures have been shown to be reliable, valid, and responsive for the general U.S. population [33], people with chronic illnesses [34,35], and people with cancer [36,37].

#### 2.3.3. CAM Activities Questionnaire

We developed an Activities Questionnaire to assess other activities participants may engage in that could impact patient-reported symptom and functioning QOL outcomes, including, among others, support groups and workshops related to cancer, physical therapy, counseling, and complementary and alternative medicine (CAM) practices. We asked participants whether or not they currently do the activity (for CAM: yoga, meditation, massage, or herbal/dietary supplements), and if so, how frequently (one or more times/week, a couple times a month, once a month, less than once a month). Items were translated into Spanish and back-translated into English by two different native Spanish speakers.

#### 2.3.4. Devotional and Spiritual Practices

As noted above, to add further specificity to our analyses, and because devotional and spiritual practices are particularly salient in Latino culture, we also sought to examine the individual influence of devotional and spiritual practices over time on specific domains of symptoms and functioning among Latina breast cancer survivors. We asked if participants currently have devotional or spirituality practices and if so, to specify what type of practices and the frequency of that practice.

### 2.4. Statistical Analyses

We generated descriptive statistics to characterize variables and used analysis of variance and Pearson correlations to evaluate bivariate associations between each continuous symptom and functioning outcome (physical functioning, satisfaction with social roles, anxiety, depression, and fatigue) and demographic and clinical characteristics. Variables related to our symptom and functioning outcomes at a level of *p* ≤ 0.10 in the bivariate analyses were then included in the regression models. We conducted a series of multiple linear regression analyses using generalized linear models for each outcome. We examined the relationship of each variable with outcomes using the least square means and beta coefficients, which represent the adjusted mean difference between groups of each covariate. All analyses we conducted using SAS version 9.3 (Cary, NC, USA).

## 3. Results

### 3.1. Sample Characteristics

#### 3.1.1. Demographics

A total of 136 survivors completed baseline assessment (see Table 2), out of which 58 survivors out of 65 randomized to the usual-care condition from the parent study completed a follow-up survey. Latina survivors were an average of 53.2 years old (11.1 SD; Range 29–83 years) and 3.3 years from the time of diagnosis (SD = 4.6 years; Range 0.06–32.0 years). Most participants (93%) were immigrants to the United States and 80% reported living in the United States 11 years or more. Survivors represented over 12 different countries and regions of origin, with 25% from Mexico, 23% from Central America, 26% from South America, 19% from the Caribbean, and 7% from the U.S. The vast majority of survivor baseline surveys were conducted in Spanish (93%) and many survivors self-identified as more than one race (43%). Just over half were married or living with a partner (57%). Many women were uncertain of the stage of their breast cancer at diagnosis (57%), or were at Stage 0 or 1 (14%). We conservatively classified unknown stage of disease as early stage 0 or 1 for the present analyses. Almost two-thirds reported having less than or equal to a high school education (62%). Treatment types included surgery (75%), chemotherapy (55%), hormonal therapies (38%), and radiation (45%).

#### 3.1.2. CAM Activities

Overall 35% (*n* = 47) of participants endorsed using CAM exclusive of devotional and spiritual practices at baseline, and within this group nearly all (*n* = 43) specified using some combination of yoga (*n* = 28), meditation (*n* = 12), massages (*n* = 8), and/or herbal supplements (*n* = 11). At follow-up 38% (*n* = 22) endorsed using CAM exclusive of devotional and spiritual practices, 20 of whom specified using some combination of yoga, meditation, massages, and/or herbal supplements.

#### 3.1.3. Devotional and Spiritual Practices

At baseline, 80% of survivors indicated using devotional and spiritual practices, including church attendance (70%) and prayer/prayer or religious groups/bible study (21%). At follow-up, 81% of survivors indicated using devotional and spiritual practices, including church attendance (74%) and prayer/prayer or religious groups/bible study (26%).

### 3.2. Baseline Cross-Sectional Analysis

Our baseline cross-sectional analyses include 136 Latina survivors; we examined the associations between baseline CAM and devotional and spiritual practices and each symptom and function outcome at baseline (see Table 3).

#### 3.2.1. Physical Function

At baseline, bivariate analyses revealed significant associations between physical function scores and patient income, marital status, stage, employment, insurance coverage, years in the U.S., and CAM use (*p* ≤ 0.01) and time since diagnosis (*p* < 0.05). Use of devotional and spiritual practice at baseline was not associated with physical function at baseline. In multivariate analyses, controlling for the above-noted demographic and clinical variables identified in bivariate analyses, survivors who did not report CAM at baseline had statistically lower physical function at baseline than those who do practice CAM (β = 3.48, *p* < 0.05).

#### 3.2.2. Satisfaction with Participation in Social Roles

In bivariate analyses, we observed significant associations at baseline between satisfaction with participation in social roles and patient employment (*p* < 0.01), chemotherapy treatment, participation in cancer-related support groups, and CAM use (*p* < 0.1). After controlling for these demographic and clinical variables in the multivariate regression model, we found no statistically significant associations between satisfaction with participation in social roles at baseline and use of any CAM modalities. Likewise, use of devotional and spiritual practices at baseline was not associated with satisfaction with social roles at baseline.

#### 3.2.3. Emotional Distress: Anxiety

Lower anxiety was significantly associated at the bivariate level with greater time since diagnosis and more years in the U.S. (*p* < 0.05), not having hormonal treatment, use of CAM as well as spiritual and devotional practices at baseline (*p* < 0.1). The relationship between baseline CAM use and baseline levels of anxiety became nonsignificant in multivariate analyses after controlling for the above-noted demographic and clinical variables. Similarly, use of devotional and spiritual practices at baseline was not associated with anxiety at baseline.

#### 3.2.4. Emotional Distress: Depression

Bivariate analyses showed a significant association between lower depression scores and greater time since diagnosis, being employed, having had hormone and radiation therapy, and use of CAM (*p* ≤ 0.05). Survivors’ reports of devotional and spiritual practices at baseline were not associated with depression at baseline. After controlling for the above demographic and clinical variables in the multivariate analyses, CAM use at baseline remained significantly associated with lower baseline depression scores (β = −5.49, *p* < 0.01).

#### 3.2.5. Fatigue

At the bivariate level, higher fatigue scores at baseline were significantly associated with older age, not being employed, and having had chemotherapy (*p* < 0.05). Neither CAM use nor use of devotional and spiritual practices at baseline were significantly associated with fatigue scores at baseline; thus we did not conduct multivariate analyses.

### 3.3. Longitudinal Analysis

Longitudinal analyses included the 58 Latina survivors who completed a follow-up assessment. We examined the impact of baseline CAM and devotional/spirituality practices separately on each symptom and function outcome, controlling for baseline level of the outcome (see Table 3). We also explored cross-sectional associations between follow-up CAM and follow-up outcomes. Finally, we examined whether CAM and devotional/spirituality practices were associated with changes between baseline and follow-up in Latina survivors’ scores on the functioning and symptom outcomes.

#### 3.3.1. Physical Function

In bivariate analyses, we found significant associations between better physical functioning at follow-up and survivors’ reports of being married, having higher income, insurance, lower stage at diagnosis, longer time since diagnosis and using CAM at baseline (*p* ≤ 0.05). After controlling for baseline level of physical functioning and the above demographic and clinical variables in multivariate analyses, at follow-up CAM use at baseline remained significantly associated with better physical functioning at follow-up (β = 3.48, *p* < 0.05). There was no significant association between baseline devotional/spiritual practices and physical functioning at follow-up. In contrast, we did find that survivors who reported CAM use at follow-up reported statistically *lower* physical function than those who did not practice CAM at follow-up (β = 4.19, *p* < 0.05). Neither CAM nor devotional/spiritual practices were associated with the physical functioning change score between baseline and follow-up.

#### 3.3.2. Satisfaction with Participation in Social Roles

In bivariate analyses, longer time since diagnosis, greater number of years in the U.S., baseline CAM use, and baseline devotional and spiritual practices were significantly associated with greater satisfaction with social roles at follow-up (*p* ≤ 0.05). Multivariate analyses examining CAM use and satisfaction with social roles at follow-up revealed distinct relationships. Controlling for baseline satisfaction with social roles and relevant demographic and clinical factors, CAM use at baseline was not associated with satisfaction with social roles at follow-up. Contrary to expectations, engaging in CAM-related activities at follow-up was associated with decreased satisfaction in social roles among Latina survivors, (β = −6.56, *p* < 0.01). Baseline use of devotional and spiritual practices did not remain a significant predictor of satisfaction with social roles in multivariate analyses.

We found similar results in our analyses related to change over time (difference between baseline and follow up scores on Satisfaction with Participation in Social Roles) with less improvement among patients who practiced CAM in general (β = −6.97, *p* < 0.01), and yoga specifically (β = −7.5, *p* < 0.01), compared to patients who did not practice CAM or yoga, respectively.

#### 3.3.3. Emotional Distress: Anxiety

While CAM at baseline was not associated with anxiety at follow-up at the bivariate level, insurance coverage and devotional and spiritual practices at baseline and follow-up (*p* ≤ 0.05), specifically use of prayer at baseline and church attendance at follow-up, were all significantly associated with anxiety at follow-up. After controlling for baseline level of anxiety, multivariate analysis indicated that patients who reported using prayer at baseline reported significantly less anxiety at follow-up, (β = −7.6, *p* < 0.05). Devotional/spiritual practices at baseline (β = −7.09, *p* < 0.05) and follow-up (β = −6.22, *p* < 0.05) were significantly associated with lower anxiety at follow-up. Although CAM was not associated with the anxiety change score between baseline and follow-up, devotional/spiritual practices at both baseline (β = −7.36, *p* < 0.05) and follow-up (β = −7.51, *p* < 0.05) were significantly associated with the anxiety change score between baseline and follow-up in the direction of reduced anxiety over time.

#### 3.3.4. Emotional Distress: Depression

CAM at baseline was not associated with depression at follow-up in bivariate analyses. Devotional and spiritual practices at baseline were associated with lower depression at follow-up in bivariate analyses (*p* < 0.05). In multivariate analyses controlling for baseline level of depression and relevant demographic and clinical variables, devotional and spiritual practices at baseline remained a significant predictor of depression at follow-up (β = −7.69, *p* < 0.05). Likewise, use of devotional and spiritual practices at follow-up was also significantly associated with depression at follow-up (β = −6.96, *p* < 0.05). These results were further confirmed with analyses evaluating depression change scores: use of devotional and spiritual practices at follow-up was significantly associated with positive changes (less depression) in depression change scores from baseline to follow-up (β = −6.6, *p* < 0.05).

#### 3.3.5. Fatigue

CAM activities were unrelated to fatigue at baseline or follow-up in bivariate or multivariate analyses. In bivariate analyses, use of devotional and spiritual practices at baseline was associated with lower fatigue at follow-up (*p* < 0.1). The relationship between devotional and spiritual practices at baseline remained significantly associated with lower fatigue scores at follow-up in multivariate analyses, controlling for baseline fatigue and other relevant clinical and demographic variables (β = −7.74, *p* < 0.05). Neither CAM nor devotional/spiritual practices were associated with the fatigue change score between baseline and follow-up.

## 4. Discussion

We examined CAM use and devotional and spiritual practices and patient-reported symptoms and functioning outcomes over time in a diverse sample of Latina breast cancer survivors. Results indicate a modest to high use of CAM in this sample, with about one-third reporting use of CAM activities such as yoga, meditation, massage, or herbal/dietary supplements. The majority of Latina breast cancer survivors endorsed use of devotional and spiritual practices; this high rate of devotional and spiritual practices among Latino cancer survivors is similar to high rates reported in prior research [20,38,39,40]. Recent systematic and meta-analytic reviews highlight the contributions of spiritual and religious practices to well-being among cancer survivors, with particular emphasis on finding meaning/peace and ability to make sense of illness (e.g., sense of coherence) [41,42]. In the present study, Latina breast cancer survivors who reported use of devotional and spiritual practices had lower anxiety, fatigue, and depression over time. Perhaps for some of these survivors, the ability to connect with a higher power provides comfort by fostering a perceived closer connection with God [39]. Results support the need for future research to explore the psychological and social mechanisms by which devotional and spirituality practices reduce anxiety over time among Latina breast cancer survivors. We did not explore how use of these practices related to finding meaning or a survivor’s ability to make sense of the illness experience. As prior evidence suggests, these meaning-finding elements may have an important role in adjustment to cancer [42]. Future work can examine how patients’ reports of using devotional and spiritual practices relate to greater sense of meaning and patient-reported outcomes.

Our results contribute to the small and growing literature on CAM use among cancer survivors by providing some of the first longitudinal outcomes of CAM use among Latina survivors. We observed interesting and distinct patterns of how CAM relates to patient-reported outcomes over time. CAM use demonstrated a positive association with Latina survivors’ reports of better physical functioning and lower depression over time. In contrast, among women who reported using CAM at follow-up, initial CAM use was not associated with higher satisfaction with social roles at follow up, and CAM use at the time of follow-up appeared to be related to lower levels of satisfaction with social roles and lower physical function. These distinct patterns between CAM use and satisfaction with social roles and physical function suggest possible differential functions of CAM use. Over time, CAM may have a beneficial impact on satisfaction with social roles and physical function, perhaps by expanding a breast cancer survivors’ social network with shared CAM activities (e.g., yoga classes) or providing self-care that yields increased physical well-being and greater social role satisfaction. Conversely, women who are currently experiencing less satisfaction with social roles and physical function may seek out additional CAM activities — as represented by the inverse association we observed at follow up with current CAM use and current satisfaction with social roles and physical function.

To our knowledge, this study presents one of the few longitudinal assessments of CAM use over time in a sample of Latina breast cancer survivors. Given the relatively small sample size at follow-up, our initial longitudinal results should be explored in future work with larger samples and longer follow-up assessments. Results suggest the possibility that CAM plays a complex role in symptoms and functioning outcomes for Latina breast cancer survivors. As also identified in other work, CAM use may be associated with increased psychosocial distress for some breast cancer survivors [43]. Future work can evaluate nuances of the use and benefits of CAM, including the impact of CAM and specific spirituality and devotional practices on helping survivors to make meaning and clarify a sense of purpose following a breast cancer diagnosis [42]. Future research can also explore additional CAM modalities and their use among Latina breast cancer survivors, such as Qigong, acupuncture, and others, and explore differences in outcomes based on modality type. Methodologically strong RCTs may help determine the efficacy of specific CAM modalities and their role in improving patient-reported outcomes among Latina breast cancer patients. These findings contribute to the literature on longitudinal CAM use and associations with QOL outcomes among Latina breast cancer survivors.

## 5. Conclusions

Subgroups of Latina breast cancer survivors report CAM use and many more report use of devotional and spiritual practices over time. Understanding the impact of CAM and devotional and spiritual practices on symptom and functioning outcomes (in particular, anxiety, depression and fatigue) can guide intervention design. The present results can be evaluated among larger and even more heterogeneous samples of Latina breast cancer survivors to identify similarities and differences based on cultural influences and availability of CAM services. The robust association between use of devotional and spiritual practices and reduced anxiety among Latina survivors can be used as a platform for anxiety-reduction interventions among this less-well studied group of breast cancer survivors.

## Figures and Tables

**Table 1:** Measures and Sample Questions.

Baseline and Follow-Up Measures	Selected Sample Questions
Demographics and Clinical	Education, income, insurance, breast cancer stage, age, time since diagnosis, employment, marital status.
PROMIS Emotional Distress: Anxiety (6a)	My worries overwhelmed me.
PROMIS Emotional Distress: Depression (6a)	I felt hopeless.
PROMIS Physical Function (6b)	Are you able to do chores such as vacuuming or yard work?
PROMIS Fatigue (6a)	I have trouble starting things because I am tired.
PROMIS Satisfaction with Participation in Social Roles(6a)	I am satisfied with my ability to meet the needs of those who depend on me.
CAM Activities Questionnaire Mind-Body Modalities (yoga, massage, meditation) Herbal/Dietary Practices	Do you practice any complementary and alternative medicine (for example, yoga, massage, meditation, herbal / dietary practices)? If yes, how often? If yes, please describe:
Spirituality/Devotional Practices	Do you currently have spirituality or devotional practices? If yes, how often?

**Table 2:** Baseline and follow-up demographics, complementary and alternative medicine (CAM), devotional/spiritual practices, and survivors’ symptoms and functioning.

	Baseline		Baseline		Follow-Up	
	All		(UC Only)		(UC Only)	
	*N*	%	*n*	%	*n*	%
**All**	136	100	65	100	58	100
**Time Since Dx (in Years)**		**Mean ± SD**		**Mean ± SD**		**Mean ± SD**
	132	3.31 ± 4.60	64	3.23 ± 4.21	57	3.42 ± 4.40
**Survey Language**						
English	10	7	5	8	3	5
Spanish	126	93	60	92	55	95
**Race**						
Black or African American	8	6	5	8	5	9
White or Caucasian	39	29	17	26	15	26
More than One Race	59	43	31	48	29	50
Don’t Know/Unsure/Prefer Not to Answer	30	22	12	18	9	16
**Marital Status**						
Single/Separated/Divorced/Widowed	59	43	27	42	22	38
Married/Living with a Partner	77	57	38	58	36	62
**Age at Diagnosis**						
<50	75	55	36	55	33	57
50+	61	45	29	45	25	43
**Age at Survey Completion**						
Missing	2	1	1	2	1	2
<50	52	38	26	40	23	40
50+	82	60	38	58	34	59
**Stage**						
Stage 0/I/Unknown	96	71	48	74	46	79
Stage II	22	16	9	14	7	12
Stage III/IV	18	13	8	12	5	9
**Education**						
≤High school diploma, or GED	84	62	37	57	34	59
>High school	52	38	28	43	24	41
**Employment**						
Working	44	32	20	31	20	34
Not working	92	68	45	69	38	66
**Income**						
<$30,000 (includes prefer not to answer/unsure *n* = 43)	105	77	51	78	45	78
>$30,000	31	23	14	22	13	22
**Insurance**						
Group Insurance/Private/Non-government	27	20	14	22	13	22
Government (includes Don’t know/Unsure *n* = (26)	109	80	51	78	45	78
**Years in the US**						
Missing	5	4	2	3	2	3
1–10 years	22	16	12	18	11	19
11+ years	109	80	51	78	45	78
**Surgery**						
No	34	25	16	25	14	24
Yes, Any surgery	102	75	49	75	44	76
Yes, Mastectomy	71	52	27	42	19	41
Yes, Lumpectomy	30	22	15	23	9	20
**Chemotherapy**						
No	61	45	26	40	25	43
Yes	75	55	39	60	33	57
**Hormonal Therapy**						
No	84	62	40	62	35	60
Yes	52	38	25	38	23	40
**Radiation Therapy**						
No	75	55	35	54	31	53
Yes	61	45	30	46	27	47
**Complementary & Alternative Medicine Use Baseline**						
Yes, CAM, Overall	47	35	24	37	21	36
CAM Overall, One or more times per week	22	16	11	17	9	16
CAM Overall, < One or more times/month	25	18	13	20	12	21
Yes, CAM, Yoga/Meditation/Massages/Herbal Supplements	43	32	21	32	18	31
**Devotional and Spiritual Practices Baseline**						
Yes, Devotional/Spiritual Practices, Overall	109	80	54	83	49	84
D/S * Overall, 1 or more times/week	86	63	41	63	36	62
D/S Overall, <One or more times/month	23	17	13	20	13	22
Yes, Church	95	70	44	68	40	69
Yes, Prayer/Religious Groups/Bible Study	30	22	18	28	16	28
**Complementary & Alternative Medicine and Devotional/Spiritual Practices (Post Intervention)**						
Yes, CAM, Overall			18	28	22	38
Yes, CAM, Yoga/Meditation/Massages/Herbal Supplements			17	26	20	34
Yes, Devotional/Spiritual Practices, Overall			37	57	47	81
Yes, Church			33	51	43	74
Yes, Prayer/Prayer or Religious Groups/Bible Study			10	15	15	26
**Symptoms and Functioning**	**Baseline**		**Baseline**		**Follow-Up**	
	**All**		**(UC Only)**		**(UC Only)**	
Baseline Physical Functioning Mean ± SD	44.28 ± 9.34		44.33 ± 9.64		44.71± 9.91	
Baseline Satisfaction with Social Roles Mean ± SD	48.35 ± 7.65		48.99 ± 7.71		49.39 ± 6.95	
Baseline Anxiety Mean ± SD	53.63 ± 11.55		52.94 ± 9.95		52.31 ± 9.60	
Baseline Depression Mean ± SD	49.83 ± 10.97		48.22 ± 9.52		47.80 ± 9.30	
Baseline Fatigue Mean ± SD	51.41 ± 12.6		49.57 ± 11.99		49.19 ± 11.71	

* Devotional/spiritual practices. Note: We did not find any significant differences in demographic, clinical, or outcome variables when we compared the Baseline All sample (*N* = 136) to the Baseline (UC Only) sample (*n* = 65) or the Follow-Up (UC Only) sample (*n* = 58).

**Table 3:** Multivariate results.

QOL Outcomes	Baseline	Follow-up
Physical Function	CAM: β = 3.48, *p* < 0.05	Baseline CAM β = 3.48, *p* < 0.05
	D/S *: *p* > 0.05	Baseline D/S: *p* > 0.05
Satisfaction with Social Roles	CAM: *p* > 0.05	Baseline CAM: *p* > 0.05
	D/S: *p* > 0.05	Baseline D/S: *p* > 0.05
Anxiety	CAM: *p* > 0.05	Baseline CAM: *p* > 0.05
	D/S: *p* > 0.05	Baseline D/S: β = 6.48, *p* < 0.05
Depression	CAM: β = −5.49, *p* < 0.01	Baseline CAM: *p* > 0.05
	D/S: *p* > 0.05	Baseline D/S: β = 7.69, *p* < 0.05
Fatigue	CAM: *p* > 0.05	Baseline CAM: *p* > 0.05
	D/S: *p* > 0.05	Baseline D/S β = −7.12, *p* < 0.05

Note: Follow-up was conducted approximately 4 months after baseline. Table does not include cross-sectional outcomes between CAM or devotional / spiritual practices use at follow-up and symptoms and function at follow-up. * Devotional and Spiritual Practices.
